# Acoustic Emission Monitoring of High-Strength Concrete Columns Subjected to Compressive Axial Loading

**DOI:** 10.3390/ma13143114

**Published:** 2020-07-13

**Authors:** Rami Eid, Boris Muravin, Konstantin Kovler

**Affiliations:** 1 Civil Engineering Department, SCE—Shamoon College of Engineering, Beer Sheva 8410802, Israel; 2 Integrity Diagnostics Ltd., Netanya 4250407, Israel; boris@ndt.co.il; 3 Faculty of Civil and Environmental Engineering, Technion—Israel Institute of Technology, Haifa 3200003, Israel; cvrkost@technion.ac.il

**Keywords:** reinforced-concrete, acoustic emission, AE, columns, high-strength concrete, HSC, transverse steel reinforcement, confinement

## Abstract

Acoustic Emission (AE) nondestructive tests have attracted great interest for their use in the determination of structural properties and behavior of reinforced concrete (RC) elements. One of the applications this method can contribute to is in high-strength concrete (HSC) columns. These elements have a great advantage in the lower stories of high-rise buildings. However, the premature failure of the concrete cover and the brittleness nature of the failure is of a concern for engineers. This paper presents a study on the AE monitoring of HSC columns subjected to compressive axial loading. The study consists of four large-scale reinforced HSC columns with different confinement reinforcement and height. It is shown that the AE distributions in the columns are categorized by three stages. Moreover, the levels of loads reached at the first AE macro event are similar to the lower range levels of the nominal axial compressive strengths of the tested specimens, while the majority of macro AE events are located at the concrete cover. Based on the results of this study, AE monitoring can provide indications for the damage and load levels attained by reinforced high-strength concrete columns subjected to compressive axial loading.

## 1. Introduction

The use of high-strength concrete (HSC) for the lower story columns in high-rise buildings is very attractive for architects and engineers [1]. One of the main concerns of using HSC for such critical structural elements is the brittleness nature of the failure mode. Confinement through transverse steel reinforcement (TSR) can transfer the failure mode to a more ductile one; however, the failure of the column’s concrete cover cannot be prevented by the confinement. Spalling off the concrete cover occurs prematurely, i.e., before the concrete reaches its full compressive strength (derived from tests of standard cylinders—150 mm diameter by 300 mm height) [2,3]. It should be noted that concrete spalling can result also from exposure to fire [4,5]. This phenomenon is dominant in HSC and one of the protection methods is the addition of polypropylene fibers to the concrete mixture. These fibers melt during the exposure to fire allowing the release of the steam pressure, thus reducing the risk of concrete spalling [6]. Nondestructive tests (NDT) methods based on acoustic, electromagnetic, thermographic, and optical phenomena are becoming more popular for determining several structural properties and behavior of reinforced concrete (RC) elements [7,8,9]. For example, thermographic testing can monitor the temperatures which can influence the reinforcement corrosion in concrete [8]. Electromagnetic NDT can be used to detect location and diameter of reinforcement bars in RC elements and fiber spacing in steel fiber-reinforced concrete elements [9,10]. Ultrasonic methods are used to detect concrete cracking due to steel reinforcement corrosion [11,12].

The Acoustic Emission (AE) nondestructive test method was used by several research studies to examine the failure mode and the development of cracks in structural RC elements [13,14]. Studies have investigated the use of AE technique on normal strength concrete (NSC) columns cast in stay-in-place fiber-reinforced polymer (FRP) tubes [15] or wrapped with FRP [16]. These studies showed that the cumulative AE counts can indicate the stage of damage [15] and provides a better understanding of the crack process of FRP-confined NSC columns [16]. Puri and Weiss [17] performed axial compressive loading and unloading tests on NSC cylinders (76 mm diameter by 406 mm height). They found a linear relationship between the AE energy and the dissipated fracture energy, and thus the AE energy can be used to evaluate the column’s damage. Other studies [18,19] found a high correlation between the AE-strain hysteretic energies of RC beam-column connections and slabs under simulated earthquake loadings. These studies proposed formulas based on the recorded AE to predict the damage level for the examined RC elements. Moreover, AE signals were used to analyze the damage of confined circular concrete-filled steel tubular (CCFT) columns [20] and FRP-CCFT columns and showed the possibility of AE to predict initial steel yielding and to provide failure warning [21]. The AE technique was also implemented for RC beams and revealed that AE parameters increased with increasing beam thickness [22]. The present study examines the damage levels of HSC-reinforced columns subjected to axial compressive loading. Moreover, the study aims to investigate the premature failure of HSC cover in RC circular columns. This premature failure is the main reason of the higher safety factors used in the design of reinforced HSC compared to NSC columns [1,23,24,25].

## 2. Experimental Program

**Figure 1:**
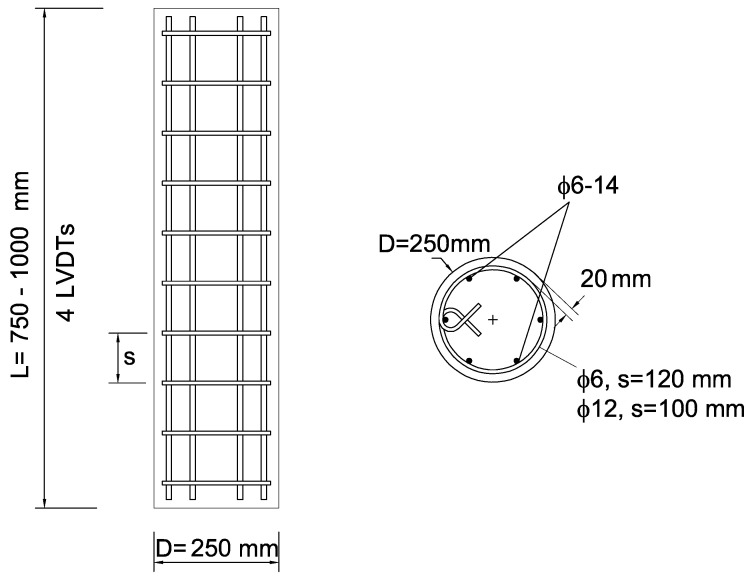
Reinforcement cage properties of tested columns.

An experimental program was designed and performed to investigate the effectiveness of the AE technique for monitoring the cracking development and the damage levels of HSC columns. The experimental program included four circular reinforced HSC column specimens of 250mm diameter. The test variables are as follows. The height of the columns are H=750 and 1000 mm, and the volumetric transverse reinforcement ratios are $\rho_{s}=0.44$ and 2.03%. These variables were chosen in order to examine their influence on the damage levels and the cracking development monitored by the AE technique in HSC columns subjected to axial compressive loading. The specimens are defined by the TSR hoops bar diameter (6 and 12 mm) and spacing (120 and 100 mm) and by the height of the specimen (*S* for H=750 mm and *L* for H=1000 mm). Details of the specimens tested at the National Building Research Institute laboratory at the Technion—Israel Institute of Technology are presented in Table 1 and Figure 1.

### 2.1. Material Properties

#### 2.1.1. Concrete

The target concrete unconfined strength of the designed specimens was set to 78 MPa. The concrete mixture of the specimens was prepared in the laboratory. The concrete mixture properties, which had a 0.27 water–cement ratio, are given in Table 2 [1,26]. It should be noted that the amount of polypropylene fibers used can be sufficient to minimize concrete spalling of HSC exposed to fire [4]. However, in this study, the intention of adding polypropylene fibers to the concrete mixture is to reduce the possible plastic shrinkage. Three standard concrete cylinders 150mm × 300mm (diameter × height) were tested under axial compression to derive the concrete average compressive strength at 28 days (=testing time), $f_{c}^{\prime }$ (Table 1).

#### 2.1.2. Steel Reinforcement

The longitudinal steel reinforcement consisted of six 14mm diameter deformed bars. Hoops of 6mm diameter plain bars and 12mm deformed bars were used as TSR. At least three tensile tests were performed on reinforcement bar coupons for each batch of steel to obtain the average yield strength (Table 1).

### 2.2. Test Set-Up and Instrumentation

**Figure 2:**
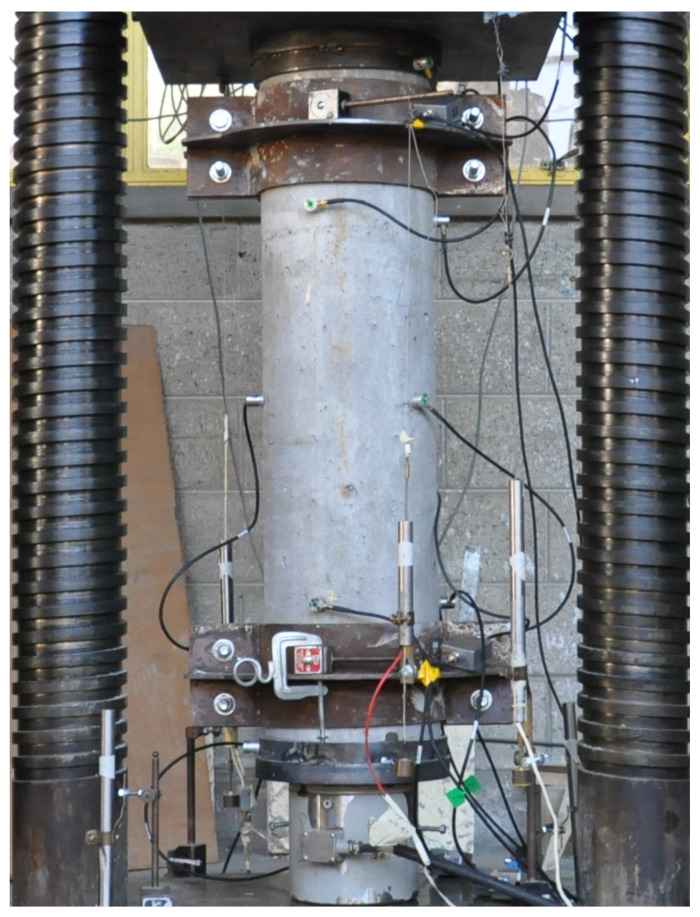
Test set-up of the reinforced concrete column specimens.

**Figure 3:**
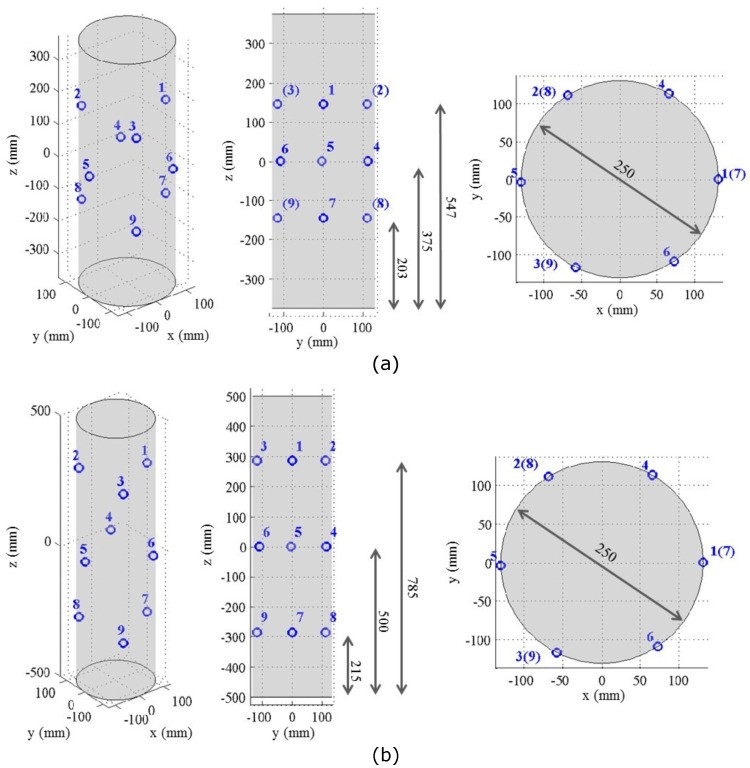
Position of Acoustic Emission (AE) sensors (**a**) specimens F6S120S and F12S100S, and (**b**) specimens F6S120L and F12S100L.

The RC columns and the concrete cylinders were cured for 7 days and after that were laid in the laboratory ambient conditions to the day of testing. Specimen axial displacement was recorded using four Linear Variable Differential Transformers (LVDTs). Two LVDTs (with a gauge length of 500mm for specimens F06S120S and F12S100S and 750mm for specimens F06S120L and F12S100L) were attached to the steel collars installed at the top and bottom of the specimens to prevent local failure, and another two LVDTs were attached to the press’s rigid steel plates (Figure 2). A load cell was used to measure the compressive load applied during the test. AE test method was applied to monitor initiation and development of damage in RC columns to failure. For this purpose, a multichannel acoustic emission system PCI8 manufactured by Mistras corporation was used. Monitoring was performed using nine 150 kHz resonant sensors mounted using cyano-acrylic adhesive and placed as specified in Figure 1, Figure 2 and Figure 3. The acoustic emission parameters of the detected and measured signals during the test included time of AE wave arrival, peak amplitude, energy, absolute energy, signal strength, rise time, duration, counts, average frequency, root mean square (RMS), and average signal level (ASL). Signal detection was performed using fixed threshold at all channels. For each detected signal, a corresponding waveform was recorded. Moreover, steel cups were used for sand capping the ends of the columns to ensure uniform distribution of the loading (Figure 2). The specimens were tested under compressive axial loading rate of 3 kN/s. The rigid hydraulic press used for testing has load-controlled capabilities and a capacity of 5000 kN (see Figure 2).

## 3. Test Results

### 3.1. General

**Figure 4:**
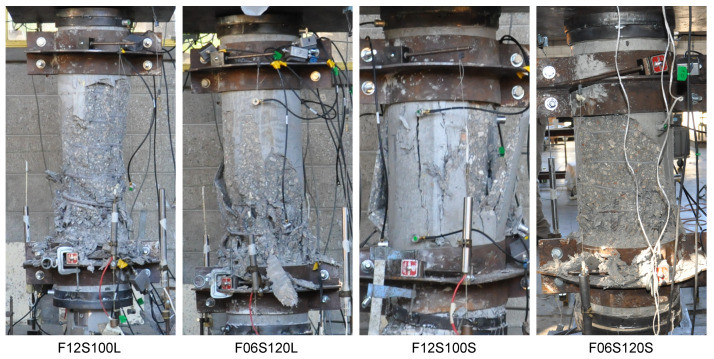
Appearance of specimens after testing.

**Figure 5:**
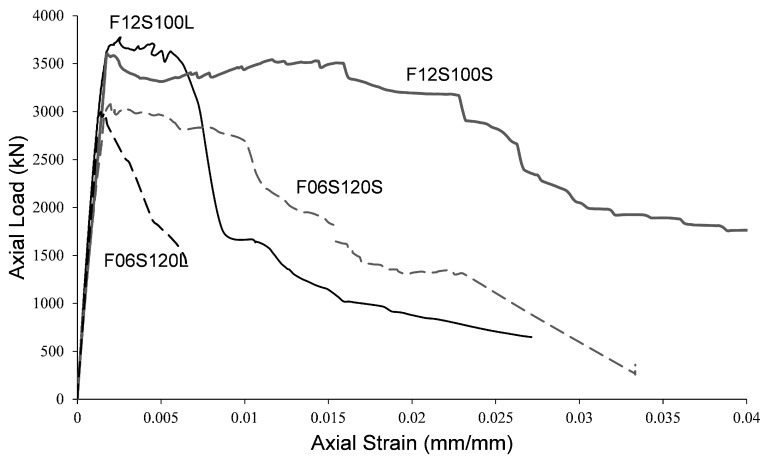
Axial load versus axial strain of the tested specimens

Figure 4 shows the appearance of the columns after testing. Figure 5 show the axial compressive load versus the axial strain (measured from the central LVDT’s) of the tested specimens. Moreover, Table 3 presents the maximum compressive axial load, $P_{max}$; its corresponding strain, $\epsilon_{c1}$; the post-peak strain at 50% of the maximum load, $\epsilon_{cp50}$; and the area under the load-strain curves, $A_{p50}$. The latter two parameters indicate the level of ductility reached by the specimens. It is shown in Figure 5 and Table 3 that, as expected, the overall behavior of specimens F12S100S and F12S100L (with higher amounts of TSR) is better than that of specimens F06S120S and F06S120L in terms of axial load capacity and ductility. It should be noted that the axial strain in Figure 5 is calculated based on the LVDTs gauge lengths equal to 500mm for the shorter specimens (F06S120S and F12S100S) and 750mm for the longer specimens (F06S120L and F12S100L). As the local failure of the specimens with a similar amount of TSR (i.e., F06S120S-F06S120L and F12S100S-F12S100L) was about the same length, different axial strain values can be obtained from the displacements recorded based on LVDTs of different gauge lengths. This fact results in the lower ductility derived for the longer specimens (F06S120L and F12S100L) compared to the shorter ones (F06S120S and F12S100S).

**Figure 6:**
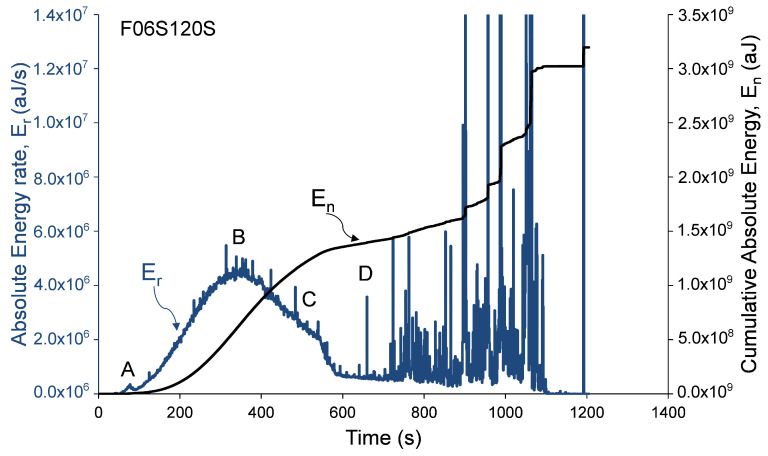
Absolute and cumulative absolute energy versus time—specimen F06S120S.

**Figure 7:**
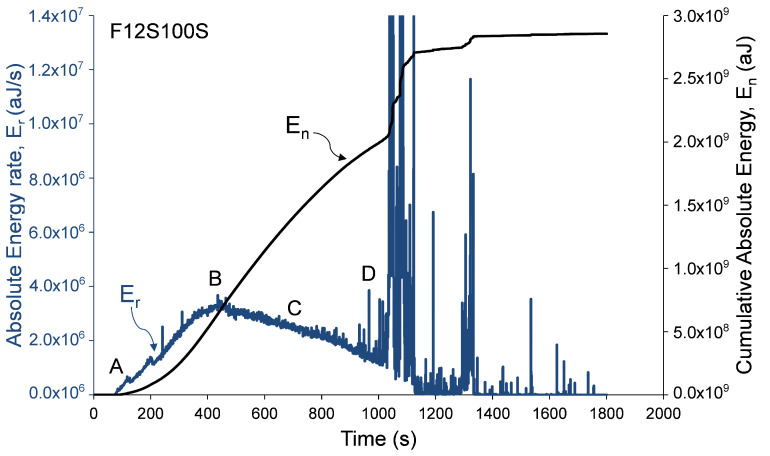
Absolute and cumulative absolute energy versus time—specimen F12S100S.

**Figure 8:**
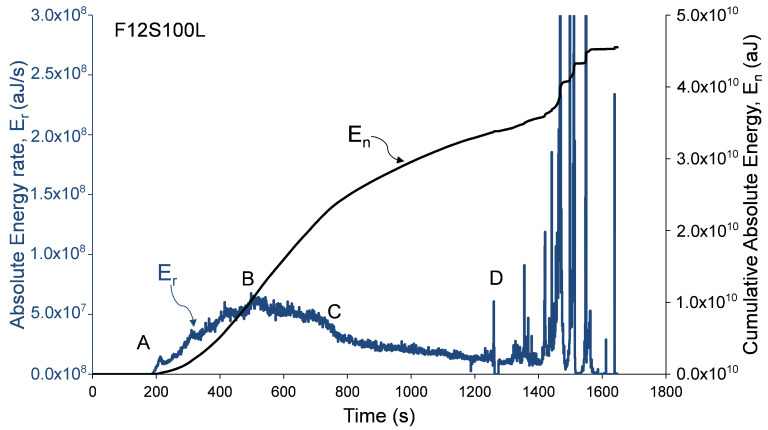
Absolute and cumulative absolute energy versus time—specimen F12S100L.

Analysis of damage development in specimens during loading was performed by calculation of distributions related to AE Absolute Energy (*E*) and specifically, by calculation of AE cumulative absolute energy, $E_{n}$, and AE absolute energy rate, $E_{r}$, shown in Figure 6, Figure 7 and Figure 8. AE absolute energy (*E*) is an AE hit parameter derived by a squared sum of AE signal’s voltage along the signal’s duration divided on input impedance (10 kΩ) and presented in attoJoules (aJ) units. It is related phenomenologically to a portion of mechanical energy released in form of AE waves during a single fracture event in the RC element during its loading. Therefore, accumulation of fracture damage and its development can be instrumentally monitored by cumulative AE absolute energy, $E_{n}$. At the same time, the rate of damage accumulation and severity of fracture events, such as crack propagation in the material, change during increasing loading and so released AE absolute energy. Therefore, in order to track changes and trends in damage development, AE absolute energy rate, $E_{r}$, was calculated as a sum of *E* of all AE events detected during time periods of 1 second. AE absolute energy rate ($E_{r}$) allows detection of short duration time trends in acoustic emission data that consist of a large number of AE signals. It should be noted that, for specimen F06S120L, the AE system stopped to save AE data after 209 seconds due to technical issue. Therefore, the AE analysis of this specimen is based on the readings that were manually taken during testing (as presented in Table 3). Data presented in Figure 6, Figure 7 and Figure 8 was recorded by sensor number 5 (see Figure 3), which was located in the middle of the specimen. Similar results were observed by all other sensors, which is reasonable taking into consideration the relatively small sensor spacing.

These figures show that AE distributions are categorized by three stages obtained in all specimens. These stages are defined by the following four reference points. (A) Initiation of AE activity, (B) peak AE absolute energy rate distribution (C) beginning of steady damage accumulation, and (D) the first AE macro event and initiation of macro-damage development. Reference points A, B, and D were obtained from AE absolute energy rate, while event C was obtained from the cumulative AE absolute energy distribution at the moment, when a principle drop in the distribution’s slope was detected. Thus, point C was determined at the intersection of two tangential lines defined along the cumulative absolute energy curve (between points B and D). Moreover, point D was determined as the first AE event with discrete burst energy that is significantly higher than the preceding stage (which is characterized by concrete micro-cracking). The recorded loads at these events are given in Table 3 for all specimens. The change in the cumulative absolute AE energy, $E_{n}$, can indicate the damage level reached by the column [15]. It should be noted that this behavior, of three damage stages, was also reported by Mirmiran et al. [15] for NSC FRP-confined columns.

### 3.2. Acoustic Emission and Compressive Behavior

**Figure 9:**
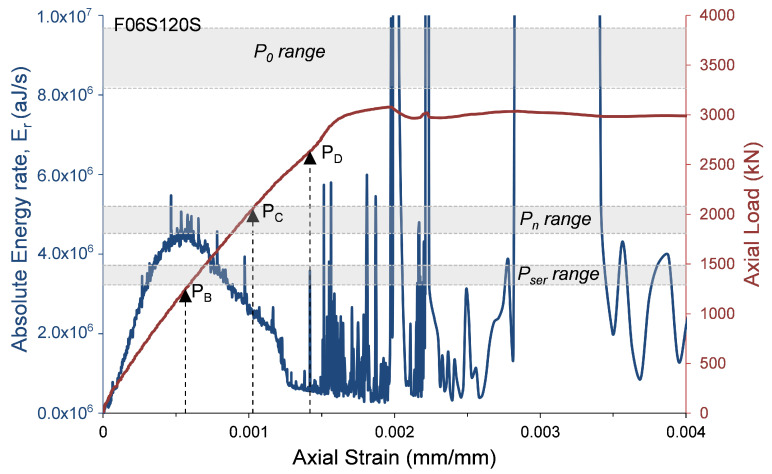
Absolute energy and axial load versus axial strain—specimen F06S120S.

**Figure 10:**
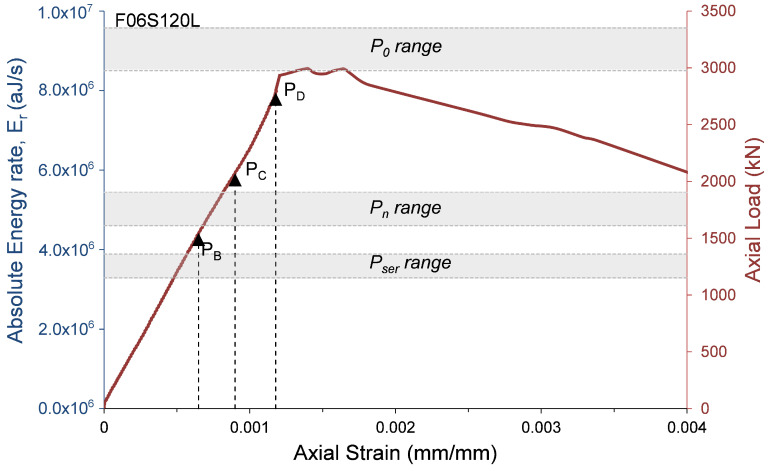
Absolute energy and axial load versus axial strain—specimen F06S120L.

**Figure 11:**
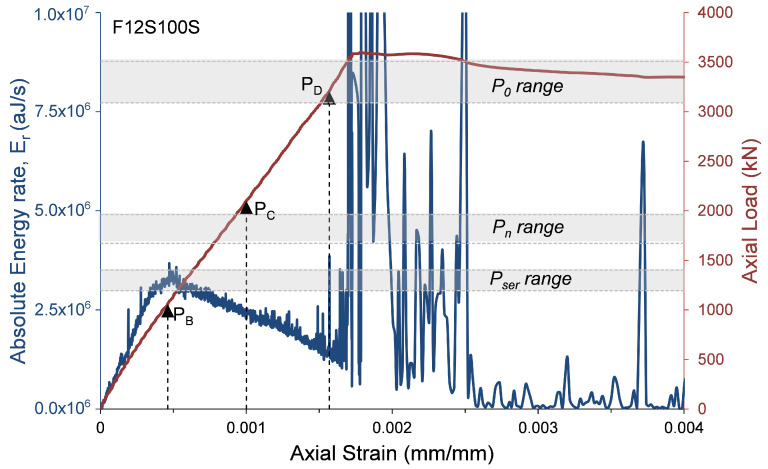
Absolute energy and axial load versus axial strain—specimen F12S100S.

**Figure 12:**
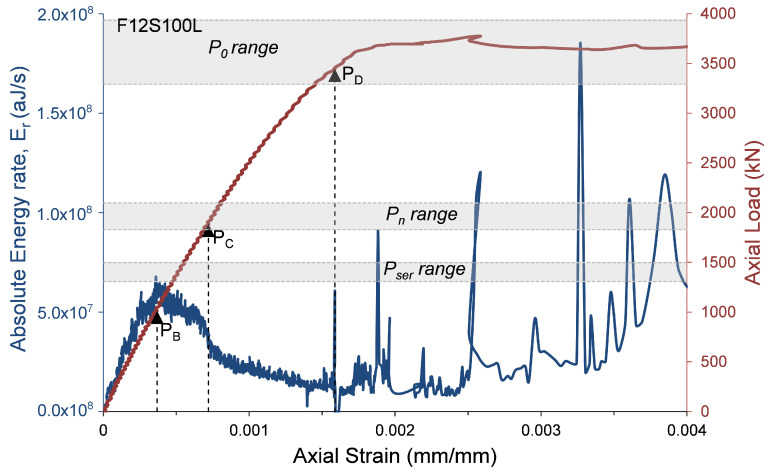
Absolute energy and axial load versus axial strain—specimen F12S100L.

The leading RC design standards [23,24,25,27] give different estimations for the nominal axial load strength of HSC columns [1]. The general expression of the nominal axial load strength is $P_{0}=\alpha_{1}\left(A_{g}-A_{s\ell }\right)f_{c}^{\prime }+A_{s\ell }f_{y}$, where $A_{g}$ is the column’s gross cross-sectional area, $A_{s\ell }$ is the longitudinal steel reinforcement cross-sectional area, $f_{y}$ is longitudinal steel reinforcement yield strength, and $\alpha_{1}$ is a parameter, which for the majority of the standards decreases as the concrete strength increases (except in the ACI [27] where $\alpha_{1}$ is constant and equal to 0.85). The $\alpha_{1}$ parameter is given as [1,23,24,25,27]

(1) $$\begin{array}{c} \alpha_{1,ACI}=0.85\\ \alpha_{1,CSA}=0.85-0.0015f_{c}^{\prime }\geq 0.67\;\;\mathrm{for}20\mathrm{MPa}<f_{c}^{\prime }\leq 80\mathrm{MPa}\\ \alpha_{1,NZS}=\left\{\begin{array}{cc} 0.85 & \mathrm{for}f_{c}^{\prime }\leq 55\mathrm{MPa}\\ 0.85-0.004\left(f_{c}^{\prime }-55\right)\geq 0.75 & \mathrm{for}55\mathrm{MPa}<f_{c}^{\prime }\leq 70\mathrm{MPa} \end{array}\right.\\ \alpha_{1,EC2}=\left\{\begin{array}{cc} \alpha_{cc} & \;\;\mathrm{for}f_{c}^{\prime }\leq 50\mathrm{MPa}\\ \alpha_{cc}\left[1.0-\left(f_{c}^{\prime }-50\right)/200\right] & \;\;\mathrm{for}50\mathrm{MPa}<f_{c}^{\prime }\leq 90\mathrm{MPa} \end{array}\right. \end{array}$$

where $\alpha_{cc}$, which has a range of 0.8 to 1.0 [23], is a coefficient that considers the long-term influence on the axial strength and of negative effects resulting from the way the load is applied [23]. One of the main reasons for decreasing $\alpha_{1}$ as the concrete strength increases is the premature failure of the concrete cover. To evaluate the AE records with the compressive behavior of the HSC specimens, the axial load and the AE absolute energy rate are presented with relation to the axial strain in Figure 9, Figure 10, Figure 11 and Figure 12. The figures also show the ranges of the following axial strengths derived from the different standards [23,24,25,27]. The nominal axial load strength, $P_{0}$; the design axial compressive strength, $P_{n}$, which is based on $P_{0}$ with material safety factors; and serviceability axial compressive strength $P_{ser}$, which is estimated as $P_{n}/1.4$, where the factor 1.4 is the equivalent safety load factor (taking into account that the dead and live loads are the dominant loads). Moreover, the loads that were reached at certain events recorded by AE sensors and were defined in Table 3 are also marked in Figure 9, Figure 10, Figure 11 and Figure 12 ($P_{B}=$ load at peak AE energy rate distribution, $P_{C}=$ load at beginning of steady damage accumulation, and $P_{D}=$ load at the first AE macro event).

It is interesting to note (Figure 9, Figure 10, Figure 11 and Figure 12) that the levels of loads reached at the peak AE absolute energy rate distribution ($P_{B}$) are similar to the levels of the assumed serviceability axial compressive strengths, $P_{ser}$, of the tested specimens. The levels of loads reached at the beginning of steady damage accumulation ($P_{C}$) are similar to the levels of the design axial compressive strengths, $P_{n}$, of the tested specimens. Moreover, and most interesting, the levels of loads reached at the first AE macro event ($P_{D}$) are similar to the lower range levels of the nominal axial compressive strengths, $P_{0}$, of the tested specimens. It is believed that the first AE macro event indicates the beginning of the premature failure of the concrete cover. Thus, from these results, it can be concluded that the lower range levels (obtained by the standards [23,24,25]) are on the safer side for determining the nominal axial compressive strength of HSC columns. This result is consistent with studies reported elsewhere [28,29,30,31,32].

**Figure 13:**
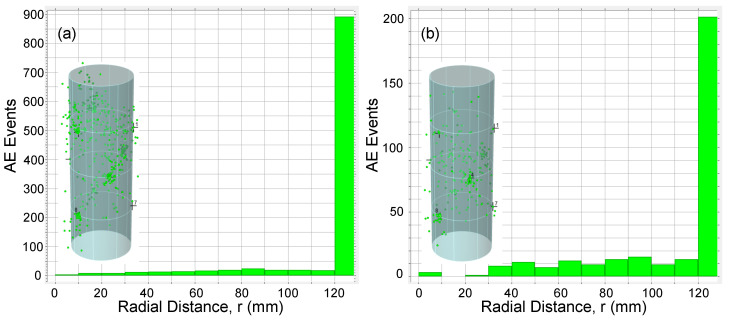
AE events along specimen F06S120S cross section for (**a**) t<500 s and (**b**) t≥500 s.

**Figure 14:**
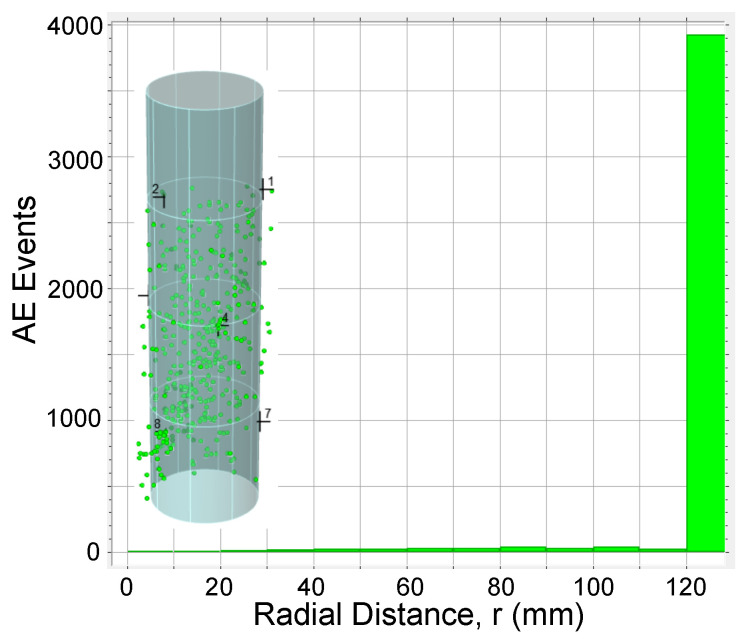
AE events along specimen F06S120L cross section for t<180 s.

**Figure 15:**
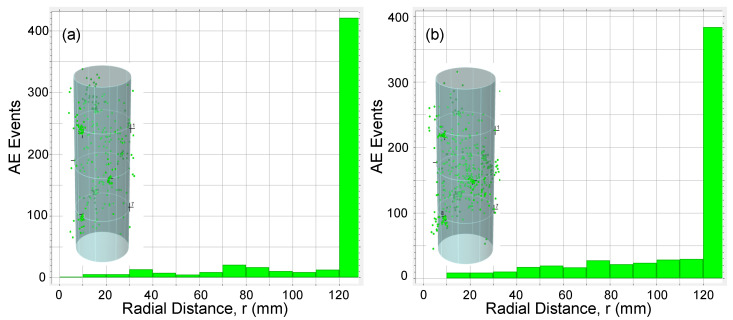
AE events along specimen F12S100L cross section for (**a**) t<722 s and (**b**) t≥722 s.

**Figure 16:**
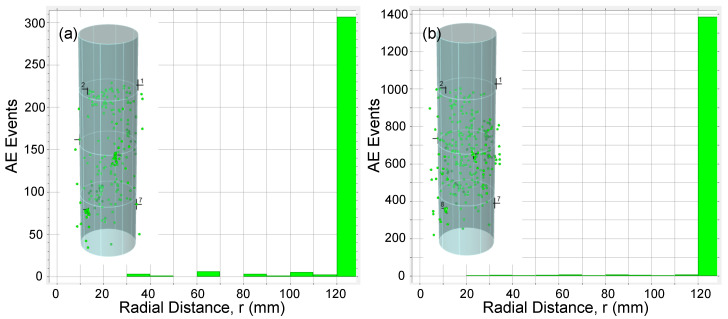
AE events along specimen F12S100L cross section for (**a**) t<750 s and (**b**) 750≤t≤950 s.

AE data was also analyzed to study 3D source location of the AE events detected during loading tests. Location calculations were performed using Vallen AE software with 3D solid algorithm [33] and using the nine AE sensors installed along the column specimens. To investigate the distribution of AE events across a cross section of specimen, a radial distance for every AE event was calculated. Figure 13, Figure 14, Figure 15 and Figure 16 show the AE source events along the radial distance of the column’s cross section (taking into account the events at the entire column’s height) for all specimens. The figures show that the majority of AE events were located at the the concrete cover and especially those associated with the macro cracking at load levels around $P_{D}$ (see Figure 13b, Figure 15b, and Figure 16b). This result conform to the phenomenon mentioned above, i.e., the premature failure of the concrete cover in HSC columns. It should be also noted that from the presented results there is a slight difference in the overall AE activity before the onset of the macro cracking events for columns with different confinement reinforcement or height.

## 4. Conclusions

This paper presents a study on the acoustic emission (AE) monitoring of high-strength concrete columns subjected to compressive axial loading. The study consists of four large-scale reinforced high-strength concrete columns with different confinement reinforcement amounts and heights. The results of the study show the following.

- The AE distributions in the columns are categorized by three stages defined by the initiation of AE activity, the peak AE absolute energy rate distribution, the beginning of steady damage accumulation, and the first AE macro event and initiation of macro-damage development.
- The levels of loads reached at the peak AE energy distribution are similar to the levels of the assumed serviceability axial compressive strengths of the tested specimens.
- The levels of loads reached at the beginning of steady damage accumulation are similar to the levels of the design axial compressive strengths of the tested specimens.
- The levels of loads reached at the first AE macro event are similar to the lower range levels of the nominal axial compressive strengths of the tested specimens.
- The majority of AE events are located at the concrete cover and especially those associated with the macro-cracking.

It is believed that the first AE macro event indicates the beginning of the premature failure of the concrete cover. Thus, this phenomenon should be taken into account in the determination of the nominal axial load compressive strength as prescribed in part of the leading standards. Moreover, based on the results of this study, AE monitoring can provide correlation between the damage and load levels (e.g., design axial compressive strength) attained by reinforced high-strength concrete columns subjected to compressive axial loading and the AE results. Future studies can include additional loading schedules like loading/unloading to provide more information regarding stages of damage/cracks development in the columns and specifically regarding the load levels at which irreversible damage is initiated.

## Figures and Tables

**Table 1:** Details of reinforced concrete specimens.

					Longitudinal Reinforcement		Transverse Reinforcement			
	Specimen No.	$f_{c}^{\prime }$ MPa	D mm	H mm	$f_{y}$ MPa	$\rho_{s\ell }$ %	$\phi_{h}$ mm	s mm	$f_{yh}$ MPa	$\rho_{s}$ %
1	F06S120S	84.8	250	750	433	1.88	6	120	288	0.44
2	F06S120L	72.1	250	1000	433	1.88	6	120	288	0.44
3	F12S100S	75.9	250	750	433	1.88	12	100	435	2.03
4	F12S100L	86.3	250	1000	433	1.88	12	100	435	2.03

**Table 2:** Properties of concrete mixture.

Variable	(kg/m${}^{3}$)
Cement	460
Fine aggregate—quartz sand	220
Intermediate aggregate (9 mm)	900
Coarse aggregate (19 mm)	550
High-Range Water Reducer	9
Fly Ash	100
Polypropylene Fibers	1
Water	150
Slump (mm)	175
Water to cementitious materials w/cm	0.27

**Table 3:** Test results.

Specimen	$P_{max}$	$\epsilon_{c1}$	$\epsilon_{cp50}$	$A_{p50}$	$P_{A}$ * $\left(P_{A}/P_{max}\right)$	$P_{B}$ ** $\left(P_{B}/P_{max}\right)$	$P_{C}{\;}^{†}$ $\left(P_{C}/P_{max}\right)$	$P_{D}{\;}^{‡}$ $\left(P_{D}/P_{max}\right)$	$P_{0,EC2}$ $\left(P_{0,EC2}/P_{max}\right)$	$P_{0,ACI}$ $\left(P_{0,ACI}/P_{max}\right)$	$P_{0,CSA}$ $\left(P_{0,CSA}/P_{max}\right)$	$P_{0,NZS}$ $\left(P_{0,NZS}/P_{max}\right)$
	**(kN)**	**(mm/mm)**	**(mm/mm)**	**(kN)**	**(kN)**	**(kN)**	**(kN)**	**(kN)**	**(kN)**	**(kN)**	**(kN)**	**(kN)**
F06S120S	3079	0.0020	0.0168	36	154	1249	2054	2636	3268	3872	3352	3463
					(0.05)	(0.41)	(0.67)	(0.86)	(1.06)	(1.26)	(1.09)	(1.12)
F06S120L	2994	0.0014	0.0065	13	89	1549	2074	2783	3026	3352	2976	3114
					(0.03)	(0.52)	(0.69)	(0.93)	(1.01)	(1.12)	(0.99)	(1.04)
F12S100S	3604	0.0018	0.0384	109	87	1057	2100	3206	3105	3507	3091	3202
					(0.02)	(0.29)	(0.58)	(0.89)	(0.86)	(0.97)	(0.86)	(0.89)
F12S100L	3774	0.0026	0.0087	30	20	1014	1889	3448	3292	3933	3395	3517
					(0.01)	(0.27)	(0.50)	(0.91)	(0.87)	(1.04)	(0.90)	(0.93)

* Load at initiation of significant AE activity. ** Load at peak AE energy distribution. ${}^{†}$ Load at beginning of steady damage accumulation. ${}^{‡}$ Load at the first AE macro event.

## Abbreviations

The following abbreviations are used in this manuscript.

AE	Acoustic Emission
TSR	Transverse steel reinforcement
NSC	Normal-strength concrete
HSC	High-strength concrete
RC	Reinforced-concrete

## References

[1] Eid R., Kovler K., David I., Khoury W., Miller S. (2018). Behavior and design of high-strength circular reinforced concrete columns subjected to axial compression. Eng. Struct..

[2] Paultre P., Khayat K.H., Langlois Y., Trudel A., Cusson D. Structural performance of some special concretes. Proceedings of the 4th International Sumposium on Utilization of High-Strength/High-performance Concrete.

[3] Foster S.J., Liu J., Sheikh S.A. (1998). Cover spalling in HSC columns loaded in concentric compression. ASCE J. Struct. Eng..

[4] Khaliq W., Kodur V. (2018). Effectiveness of polypropylene and steel fibers in enhancing fire resistance of high-strength concrete columns. ASCE J. Struct. Eng..

[5] Hager I., Mróz K. (2019). Role of polypropylene fibres in concrete spalling risk mitigation in fire and test methods of fibres effectiveness evaluation. Materials.

[6] Bangi M.R., Horiguchi T. (2012). Effect of fibre type and geometry on maximum pore pressures in fibre-reinforced high strength concrete at elevated temperatures. Cem. Concr. Res..

[7] Zhu Y.K., Tian G.Y., Lu R.S., Zhang H. (2011). A review of optical NDT technologies. Sensors.

[8] Maj M., Ubysz A., Hammadeh H., Askifi F. (2019). Non-destructive testing of technical conditions of RC industrial tall chimneys subjected to high temperature. Materials.

[9] Schabowicz K. (2019). Non-destructive testing of materials in civil engineering. Materials.

[10] Kobaka J., Katzer J., Ponikiewski T. (2019). A combined electromagnetic induction and radar-based test for quality control of steel fibre reinforced concrete. Materials.

[11] Ahn E., Kim H., Sim S.H., Shin S.W., Shin M. (2017). Principles and applications of ultrasonic-based nondestructive methods for self-healing in cementitious materials. Materials.

[12] Climent M.A., Miró M., Carbajo J., Poveda P., De Vera G., Ramis J. (2019). Use of non-linear ultrasonic techniques to detect cracks due to steel corrosion in reinforced concrete structures. Materials.

[13] Xiao M., Ju F., Ning P., Li K. (2019). Mechanical and acoustic emission behavior of gangue concrete under uniaxial compression. Materials.

[14] Abarkane C., Rescalvo F.J., Donaire-Ávila J., Galé-Lamuela D., Benavent-Climent A., Molina A.G. (2019). Temporal acoustic emission index for damage monitoring of RC structures subjected to bidirectional seismic loadings. Materials.

[15] Mirmiran A., Shahawy M., El Echary H. (1999). Acoustic emission monitoring of hybrid FRP-concrete columns. ASCE J. Eng. Mech..

[16] Ma G., Li H. (2017). Acoustic emission monitoring and damage assessment of FRP-strengthened reinforced concrete columns under cyclic loading. Constr. Build. Mater..

[17] Puri S., Weiss J. (2006). Assessment of localized damage in concrete under compression using acoustic emission. ASCE J. Mater. Civ. Eng..

[18] Benavent-Climent A., Castro E., Gallego A. (2009). AE monitoring for damage assessment of RC exterior beam-column subassemblages subjected to cyclic loading. Struct. Health Monit..

[19] Benavent-Climent A., Gallego A., Vico J.M. (2011). An acoustic emission energy index for damage evaluation of reinforced concrete slabs under seismic loads. Struct. Health Monit..

[20] Du F., Li D., Shan B., Wang Y. (2018). Failure behavior monitoring and evaluation of steel-confined reinforced concrete columns by acoustic emission under quasi-static loading. Latin Am. J. Solids Struct..

[21] Li D., Chen Z., Feng Q., Wang Y. (2015). Damage analysis of CFRP-confined circular concrete-filled steel tubular columns by acoustic emission techniques. Smart Mater. Struct..

[22] Aldahdooh M.A.A., Bunnori N.M., Johari M.M. (2013). Damage evaluation of reinforced concrete beams with varying thickness using the acoustic emission technique. Constr. Build. Mater..

[23] European Standard 1992-1-1 (2004). Eurocode 2: Design of Concrete Structures. Part 1-1: General Rules and Rules for Buildings.

[24] Canadian Standard Association (CSA) A23.3 (2014). Design of Concrete Structures.

[25] Standards New Zealand (NZS) 3101 (2006). Concrete Structures Standard, Part 1—The Design of Concrete Structures.

[26] Eid R., Cohen A., Guma R., Ifrach E., Levi N., Zvi A. (2019). High-strength concrete circular columns with TRC-TSR dual internal confinement. Buildings.

[27] American Concrete Institute ACI 318 (2019). Building Code Requirements for Structural Concrete.

[28] Razvi S.R., Saatcioglu M. (1994). Strength and deformability of confined high-strength concrete columns. ACI Struct. J..

[29] Azizinamini A., Kuska S.S.B., Brungardt P., Hatfield E. (1994). Seismic Behavior of Square High-Strength Concrete Columns. ACI Struct. J..

[30] Ozbakkaloglu T., Saatcioglu M. (2004). Rectangular stress block for high-strength concrete. ACI Struct. J..

[31] Bae S., Bayrak O. Examination of stress block Parameters for high-strength concrete in the context of ACI 318 Code. Proceedings of the Reinforced Concrete Columns with High Strength Concrete and Steel Reinforcement, SP-293.

[32] American Concrete Institute ACI 441.1R-18 (2018). Report on Equivalent Rectangular Concrete Stress Block and Transverse Reinforcement for High-Strength Concrete Columns.

[33] Vallen Systeme GmbH (2019). Vallen AE-Suite Software.

